# Prostatectomy versus definitive radiation for localized prostate cancer: revisiting the debate at a tertiary cancer center

**DOI:** 10.3389/fonc.2026.1756635

**Published:** 2026-03-02

**Authors:** Ramiz Abu-Hijlih, Abdulla Alzibdeh, Haneen Abaza, Rami Ghanem, Abdel-Hamid Khairy, Zaid Al-Kilani, Mohammad Shahait, Samer Salah, Fawzi Abuhijla, Issa Mohamad, Ayat Taqash, Hadeel Abdel Khaleq, Hikmat Abdel-Razeq, Amal Al-Omari

**Affiliations:** 1 Department of Radiation Oncology, King Hussein Cancer Center, Amman, Jordan; 2 Office of Scientific Affairs and Research, King Hussein Cancer Center, Amman, Jordan; 3 Department of Surgery, King Hussein Cancer Center, Amman, Jordan; 4 School of Medicine, Al-Sharjah University, Sharjah, United Arab Emirates; 5 Adult Oncology Department, King Fahed Specialist Hospital, Dammam, Saudi Arabia; 6 Department of Internal Medicine, King Hussein Cancer Center, Amman, Jordan

**Keywords:** Jordan, localized prostate cancer, prostatectomy, radiotherapy, recurrence

## Abstract

**Background:**

Surgery and radiotherapy are the main treatments for localized prostate cancer. Although many retrospective studies have compared their efficacy, evidence from the Middle East and North Africa (MENA) region is limited. This study reports real-world outcomes of patients treated with radical prostatectomy versus definitive radiotherapy at a tertiary cancer center in Jordan.

**Methods:**

We conducted a retrospective cohort study of patients treated between June 2006 and June 2019. Patient demographics, disease characteristics, and treatment details were extracted from electronic medical records. Primary endpoints were cancer-specific survival (CSS) and overall survival (OS); secondary endpoints included metastasis-free survival (MFS) and biochemical relapse-free survival (BRFS). Survival outcomes and group differences were evaluated with the log-rank test.

**Results:**

Among 317 patients, median age was 68 years and median PSA 12.98 ng/mL. High-risk disease predominated (62.5%), followed by intermediate (32.8%) and low-risk (4.7%). Eighty-nine patients underwent radical prostatectomy and 228 received radiotherapy with androgen deprivation therapy (ADT). Five-year CSS and OS were excellent and similar between surgery and radiotherapy (CSS: 98.7% vs. 96.7%; OS: 90% vs. 89.6%). MFS was comparable (94.9% vs. 94.2%), while BRFS was lower in the surgical group (59.8% vs. 90.5%, p < 0.001). Recurrence occurred in 44.9% of surgical versus 14% of radiotherapy patients, and prostate cancer–specific deaths were 4.4% and 4.3%, respectively.

**Conclusion:**

This study shows that surgery and radiotherapy achieve excellent long-term survival for localized prostate cancer. Radiotherapy with ADT provides superior biochemical control, emphasizing the importance of individualized, multidisciplinary treatment planning.

## Introduction

Prostate cancer ranks as the most frequently diagnosed cancer in men globally and is the second leading cause of cancer-related death (1). Nevertheless, the prognosis of localized disease remains favorable and potentially curable. Prostatectomy and definitive radiation represent the mainstay treatment options for this disease, and many reports in the literature have shed light on the advantages and complications of each approach, but outcomes are still controversial, given the heterogeneity of surgical and radiation techniques, in addition to patients and disease characteristics (2–4).

According to the American Cancer Society (ACS), Prostate-Specific Antigen (PSA) testing for men at average risk should begin at age 50 through informed and shared decision-making (5). Widespread PSA testing in several developed countries has led to a reduction in mortality, largely by increasing the detection of early-stage localized prostate cancers (6, 7). Nevertheless, a study from Jordan revealed that only 13% of older men adhered to prostate cancer screening guidelines over the past decade, and it seems that the absence of a national screening program remains a significant barrier to early detection (8, 9).

Treatment of localized prostate cancer typically ranges from active surveillance to active interventions such as surgery, radiotherapy, and androgen deprivation therapy (ADT). The treatment option is usually dictated by a multidisciplinary panel according to disease stage, risk group, performance status, and genetics, in addition to personal preferences and other factors (10). Recent years have witnessed significant advancements in treatment strategies, including the adoption of robotic surgery and the introduction of state-of-the-art radiation techniques. It is yet to be disclosed whether such a transition to more advanced treatments has influenced the oncologic outcomes in localized prostate cancer patients in a resource-limited country like Jordan.

Multiple studies in the literature have compared the oncologic outcomes of surgery and radiotherapy, and almost all of these series were retrospective in nature. To the best of our knowledge, this is the first series from the Middle East and North Africa (MENA) region comparing both modalities in localized prostate cancer. Investigating this population is particularly important given the absence of structured screening programs, the tendency for patients to present with more advanced disease, and the potential influence of a distinct genomic landscape (11–13). Moreover, it represents real-world evidence on the current practices and outcomes when compared with results from high-income countries.

This study aims to compare radical prostatectomy and definitive radiotherapy for patients with localized prostate cancer managed at a tertiary cancer center in Jordan, reporting patients and disease characteristics as well as survival outcomes.

## Methods

### Study design and population

This study is a retrospective cohort analysis of patients diagnosed with localized prostate cancer between June 2006 and June 2019 at King Hussein Cancer Center (KHCC) in Amman, Jordan. The study included patients with localized or locally advanced (node-positive) prostatic adenocarcinoma. Staging workup encompassed bone scintigraphy and computed tomography (CT) of the chest, abdomen, and pelvis. Since 2015, high-risk patients have been staged by Ga68-PSMA PET/CT, and for those with equivocal findings on conventional imaging. As per our institutional clinical practice guidelines, all patients were discussed at the genitourinary multidisciplinary tumor board before treatment initiation. This analysis included only patients who received active treatment at KHCC and had a minimum follow-up duration of three months. Patients who were referred for a second opinion and those with *de novo* metastatic disease at initial presentation were excluded.

The primary endpoints were cancer-specific survival (CSS) and overall survival (OS). While secondary endpoints were metastasis-free survival (MFS) and biochemical-relapse-free survival (BRFS). This study was conducted in accordance with the ethical principles outlined in the Declaration of Helsinki. Approval was obtained from the Institutional Review Board (IRB) at KHCC.

### Data source and collection

Patient data were retrieved from the electronic medical records and the Jordan Cancer Registry. Collected variables included date of birth, age at diagnosis, cancer stage per the American Joint Committee on Cancer (AJCC), 8th Edition (2016) (14), and risk stratification according to National Comprehensive Cancer Network (NCCN) guidelines (10).

Treatment-related variables included the primary treatment modality (radical prostatectomy or radiotherapy). Details regarding surgical approach (open versus laparoscopic) and radiation technique (3D conformal radiotherapy, intensity-modulated radiotherapy [IMRT], or volumetric modulated arc therapy [VMAT]) were captured. Additionally, margin status for surgically treated patients, androgen deprivation therapy (ADT) duration, and histopathological features such as Gleason score, perineural invasion (PNI), and lymphovascular invasion (LVI) were documented.

Outcomes related to disease progression included type of relapse, biochemical failure (defined as PSA >0.2 ng/mL following surgery as per American Urological Association (AUA)/American Society for Radiation Oncology (ASTRO)/Society of Urologic Oncology (SUO) consensus (15) or PSA nadir + 2 ng/mL following radiotherapy as per Phoenix definition (16), and the cause of death.

### Statistical analysis

Descriptive statistics were used to summarize patient demographics, clinical characteristics, and treatment modalities. Categorical variables were presented as frequencies and percentages, while continuous variables were reported as means ± standard deviation (SD) or medians with interquartile ranges (IQR). OS, CSS, BRFS, and MFS were estimated using the Kaplan-Meier method, with differences between groups assessed using the univariate non-parametric log-rank test. Stratified survival analyses were conducted based on risk groups (low, intermediate, high), nodal status (N0 versus N1), treatment modality (prostatectomy versus radiotherapy), and treatment era (based on treatment technique).

To identify factors associated with survival outcomes, multivariate Cox proportional hazards regression models were applied, with hazard ratios (HR) and 95% confidence intervals (CI) reported. A p-value of <0.05 was considered statistically significant. Data analysis was performed using SPSS version 9.4 (SAS Institute Inc, Cary, NC).

## Results

### Patient characteristics

**Figure 1:**
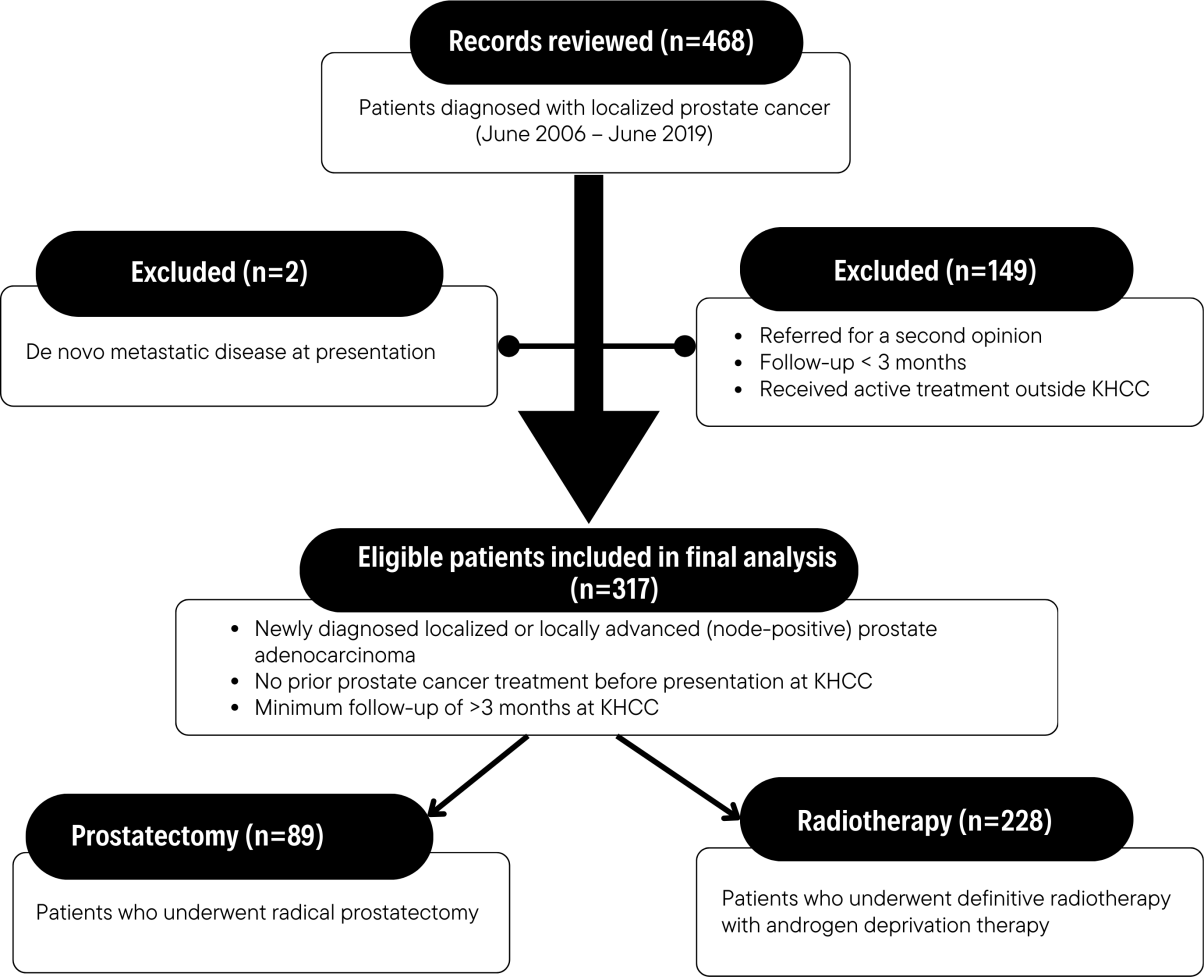
Flowchart of included prostate cancer patients diagnosed between June 2006 and June 2019.

Out of 468 reviewed consecutive patients, 317 patients with intent-to-treat were eligible at the time of final analysis. All cases diagnosed with localized prostate cancer were included in the analysis, as illustrated in the flow diagram (**Figure 1**). The median age at diagnosis was 68 years (range: 41.2–81.9), and the median PSA level was 12.98 ng/mL (range: 1.8-244). High-grade disease (Gleason 8-10) was observed in 97 cases (30.6%), with 136 (42.9%) diagnosed with AJCC 8th stage T3 and T4. On the contrary, only 12 (3.8%) were T1c. Moreover, the majority of patients 198 (62.5%) had high-risk disease; of these, 31 (9.8%) had nodal involvement (N1 disease). Additionally, 248 (78.2%) were found to have comorbidities, and 37 (11.7%) were diagnosed with second primary cancer; bladder cancer was the most frequent in 29 patients.

Among the entire cohort, 89 patients (28.1%) underwent radical prostatectomy, while 228 cases (71.9%) received radiotherapy as primary treatment. Histopathologic examination of the prostatectomy specimens revealed positive surgical margins in 32 (36%) samples, 41 (46.1%) with extracapsular extension, and 13 (14.6%) had seminal vesicle invasion. While 49 (55.1%) had PNI and 16 (18%) had LVI. Regarding treatment modality, radiation techniques evolved over the study period — transitioning from 3D conformal radiotherapy to IMRT in 2011, and later to VMAT in 2018. In addition, 83 (36.4%) patients received short-term ADT, while 160 (70.2%) were administered long-term ADT.

### Survival outcomes

The median follow-up duration was 89.5 months (range: 7.5–213 months). For the whole cohort, the primary endpoints were 5-year OS: 89.7% (95% CI: 86.0%–93.0%), and the 5-year CSS: 97.3% (95% CI: 95.1%–98.8%). While secondary endpoints reported as 5-year BRFS: 81.9% (95% CI: 77.3%–86.1%), and the 5-year MFS was 94.4% (95% CI: 91.4%–96.8%).

**Figure 2:**
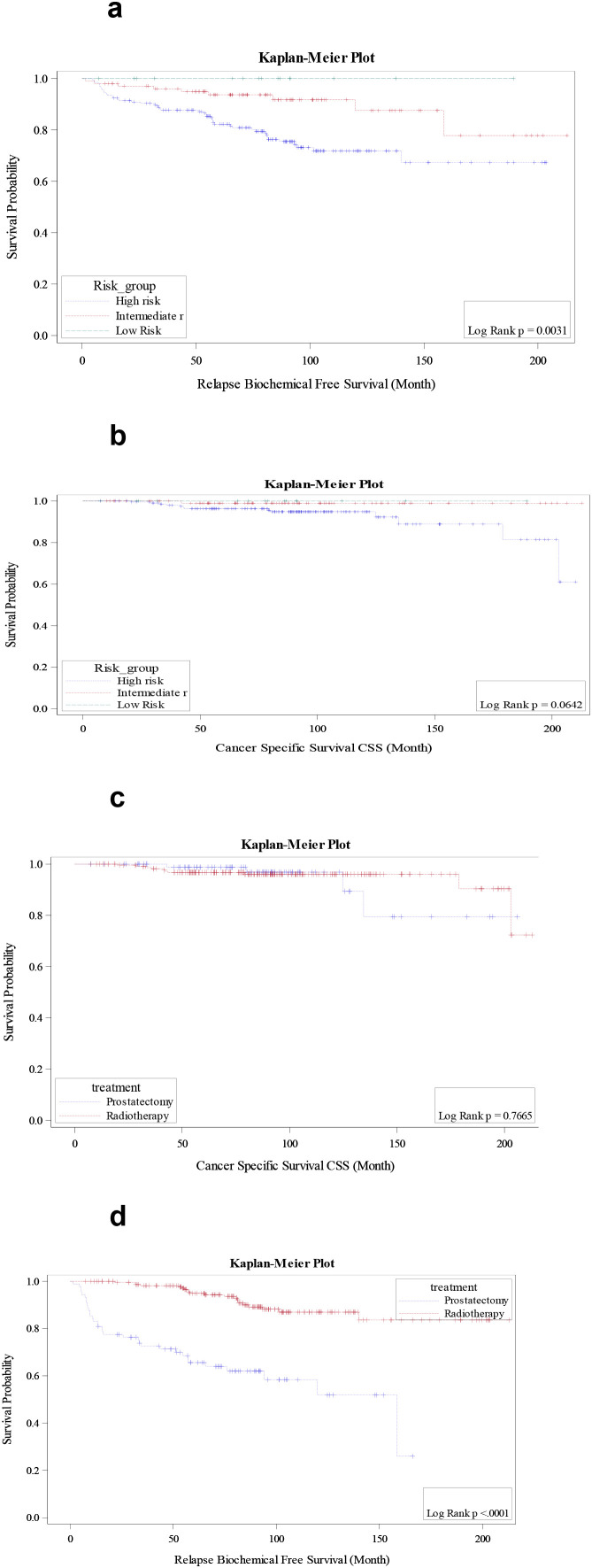
**(a)** Biochemical relapse-free survival curve according to risk groups. **(b)** Cancer-specific survival curve according to risk groups. **(c)** Cancer-specific survival curve according to treatment modality. **(d)** Biochemical relapse-free survival curve according to treatment modality.

The cumulative 5-year biochemical relapse incidence was 13.3%, while the 5-year metastatic progression incidence was 5.6%. The 5-year OS was statistically different according to risk groups, with 88.9% in high-risk, 92.0% in intermediate-risk, and 85.7% in low-risk patients (p=0.01). While the 5-year CSS was 96.3% in high-risk, 98.9% in intermediate-risk, and 100% in low-risk stage (p=0.06). Notably, the 5-year BRFS was significantly lower in the high-risk 75.3% compared to 92.6% in the intermediate-risk and 100% in the low-risk group (p<0.0031), as illustrated in **Figure 2a**. Differences in CSS across risk groups are further depicted in **Figure 2b** (p=0.064). Moreover, the 5-year MFS was 91.7% in high-risk patients, 99% in intermediate-risk, and 100% in low-risk (p=0.002). Patients with N0 disease had significantly better survival outcomes compared to N1, in terms of 5-year CSS was 98.1% compared to 89.1% in N1 patients (p=0.002), 5-year BRFS 84.7% versus 55.9% for N1 disease (p<0.001), and 5-year MFS 96.1% versus 78.6% in N1 (p<0.001), (**Supplementary Figure 5a-c**).

### Survival outcomes of surgery versus radiation

Primary endpoints were comparable between prostatectomy and radiotherapy, 5-year OS with surgery was 90% versus 89.6% in radiotherapy (*p* = 0.51), and 5-year CSS 98.7% for prostatectomy and 96.7% in radiotherapy as shown in **Figure 2c** (*p* = 0.76). As regards secondary endpoints. The 5-year BRFS was significantly lower in the prostatectomy group 59.8% compared to 90.5% in radiotherapy (*p* < 0.001) as illustrated in **Figure 2d**, while the 5-year MFS was 94.9% in the prostatectomy group and 94.2% in radiotherapy (*p* = 0.49). Interestingly, there was no statistical difference in survival outcomes based on treatment techniques (according to the year of treatment), this is shown in **Figure 6a–c**. Nevertheless, there was a trend for improvement in 5-year BRFS with changed techniques over the years, in radiotherapy, for instance, 76.2% for (2006-2011) versus 83.9% (2012-2019), (*p* = 0.27). Similarly, for surgery, 81.7% (2006-2017) versus 84.4% (2017-2019) (*p* = 0.86). At the time of last follow-up, 40 (44.9%) of patients who underwent surgery developed recurrence versus 32 (14%) in radiotherapy. Additionally, those who underwent surgery 4 (4.4%) died of prostate cancer versus 10 (4.3%) in radiotherapy. **Table 1** illustrates patients and disease characteristics in patients managed with surgery versus radiotherapy. Notably, patients with positive surgical margins had significantly worse BRFS compared with those with negative margins (38.6% vs. 71.1%; p<0.001), while no significant differences were observed in CSS (*p* = 0.10) or OS (*p* = 0.96) (**Supplementary Figures 1a–c**).

**Table 1:** Comparison of clinical details and outcomes according to treatment modality.

Characteristics	Value	Total	Treatment		P-value
			Prostatectom N=89	Radiotherapy N=228	
Age (median-years)	≤70	198 (62.5%)	81 (91.0%)	117 (51.3%)	<0.001
	>70	119 (37.5%)	8 (9.0%)	111 (48.7%)	
PSA (ng/mL)	<20	227 (71.6%)	75 (84.3%)	152 (66.7%)	0.002
	≥20	90 (28.4%)	14 (15.7%)	76 (33.3%)	
cT stage	1c	12 (3.8%)	5 (5.6%)	7 (3.1%)	0.027
	2a	19 (6.0%)	4 (4.5%)	15 (6.6%)	
	2b	75 (23.7%)	26 (29.2%)	49 (21.5%)	
	2c	75 (23.7%)	13 (14.6%)	62 (27.2%)	
	3a	68 (21.5%)	27 (30.3%)	41 (18.0%)	
	3b	61 (19.2%)	13 (14.6%)	48 (21.1%)	
	4	7 (2.2%)	1 (1.1%)	6 (2.6%)	
Nodal status	N0	286 (90.2%)	77 (86.5%)	209 (91.7%)	0.165
	N1	31 (9.8%)	12 (13.5%)	19 (8.3%)	
Gleason score	6	40 (12.6%)	14 (15.7%)	26 (11.4%)	0.073
	7	180 (56.8%)	56 (62.9%)	124 (54.4%)	
	8-10	97 (30.6%)	19 (21.3%)	78 (34.2%)	
Risk group	Low Risk	15 (4.7%)	7 (7.9%)	8 (3.5%)	0.078
	Intermediate risk	104 (32.8%)	34 (38.2%)	70 (30.7%)	
	High risk	198 (62.5%)	48 (53.9%)	150 (65.8%)	
Type of relapse	Biochemical relapse	55 (76.4%)	34 (85.0%)	21 (65.6%)	0.054
	Metastatic	17 (23.6%)	6 (15.0%)	11 (34.4%)	
Cause of death	Other causes	59 (80.8%)	12 (75.0%)	50 (83.4%)	0.491
	Prostate Cancer	14 (19.2%)	4 (25.0%)	10 (16.6%)	
Status at last follow-up	Alive	241 (76.0%)	73 (82.0%)	168 (73.7%)	0.118
	Dead	76 (24.0%)	16 (18.0%)	60 (26.3%)	

## Discussion

This study provides a comprehensive overview of the disease characteristics and treatment outcomes of localized prostate cancer in Jordan over 14 years. This relatively large cohort of 317 patients offers a comprehensive comparison of surgery and radiotherapy for localized prostate cancer. To the best of our knowledge, such a comparison from our region has not been previously reported. Furthermore, we captured multiple epidemiologic and disease-related findings. For instance, only 3.8% had T1 disease, highlighting the lack of comprehensive prostate cancer screening programs, which resulted in a more advanced stage at presentation 62.5%. The ACS recommended that men at average risk for prostate cancer engage in shared decision-making regarding PSA testing, starting at the age of 50 years (5). Meanwhile, the American Urological Association (AUA) and the U.S. Preventive Services Task Force (USPSTF) advised screening men aged 55 to 69 years based on an individual basis, but may be offered for men with certain risk factors between 40 and 54 years (14, 17). This is particularly crucial given Jordan’s evolving demographic profile, which exhibits an increasing proportion of older adults, making early detection efforts paramount (3, 11, 18).

The choice of the optimal treatment for localized prostate cancer is challenging, as it relies on several factors, including patient age, performance status, and disease grade and stage. Additional considerations, such as patient preference, available treatment techniques, and anticipated toxicity, also play a critical role. Therefore, treatment decisions are typically individualized under the umbrella of multidisciplinary discussion and the patient’s wishes. While multiple comparative studies have explored outcomes between surgery and radiotherapy, most available data originate from high-income countries, with a noticeable lack of comparative evidence from the Middle East and other LMIC settings. This gap is noteworthy, especially as molecular studies have demonstrated potential biological and genomic differences among prostate cancer populations in the region (19).

Given the available evidence, most comparative studies between surgery and radiotherapy have been retrospective analyses or meta-analyses. Only a limited number of clinical trials have attempted to evaluate both approaches in a randomized fashion to minimize bias. One example is the multicenter Swedish trial by Lennernäs et al. (20), which included men with T1–T3a disease and demonstrated comparable outcomes between the two modalities over a relatively short follow-up period (20). Another important study is the ProtecT randomized trial, which also demonstrated no significant difference in prostate cancer–specific mortality between active monitoring, surgery, and radiotherapy, although they observed higher rates of disease progression and metastasis in the active monitoring arm (2). Similarly, a Japanese trial by Akakura et al, concluded with both surgery and prostatectomy (21). A more recent study is the PACE-A, which investigated prostatectomy and stereotactic body radiotherapy (SBRT), however, only toxicity and quality-of-life data have been published thus far, while oncologic outcomes remain pending. Our results, therefore, provide comparative outcome data from a Middle Eastern cohort, contributing region-specific evidence to the limited global literature. **Table 2** compares OS and CSS events in our study to available prospective data. Notably, the CSS in our study is lower than other studies, such as ProtecT, which includes a more favorable patient cohort; nevertheless, the death rate from prostate cancer remains relatively low across different experiences and regions from the world.

**Table 2:** Summary of overall survival (OS) and cancer-specific survival (CSS) events in our cohort compared with available prospective data.

Study	Year	Type	Risk group (majority)	OS						CSS					
				Surgery			Radiation			Surgery			Radiation		
				Events	Total	%	Events	Total	%	Events	Total	%	Events	Total	%
ProtecT (3)	2016	Prospective	Early	498	553	9.9	490	545	10	548	553	1	541	545	0.7
Japanese (22)	2006	Prospective	Advanced	35	46	23.9	35	49	28.5	44	46	4.3	42	49	14.2
Swedish (20)	2015	Prospective	Early	33	45	26.6	35	44	20.4	39	45	13.3	42	44	4.5
Jordan	2025	Retrospective	Advanced	73	89	17.9	168	228	26.3	85	89	4.4	218	228	4.3

Another interesting finding was that the OS in low-risk patients was lower than in other risk groups, which might reflect the effect of competing causes of death in our population. However, the relatively small number of patients in this group might also have contributed to this observation.

With the exception of the small Japanese cohort, most patients in these prospective studies had favorable disease characteristics, primarily low- and intermediate-risk disease, which was reflected in the low number of events across these trials. In contrast, treatment of the high-risk group remains a subject of debate at the multidisciplinary level. There is high-level evidence that the addition of radiation and ADT in high-risk disease is associated with a survival benefit (23). Nevertheless, only one clinical trial tested surgery in high-risk patients, the Scandinavian SPCG-15, but the results have not yet been published (24). Despite this, surgical management continues to be broadly accepted across urology societies worldwide, as supported by population-based studies and meta-analysis reports (25). For example, Wallis et al. carried out a systematic review of 19 studies to address the lack of level 1 evidence, and found that mortality was higher with radiotherapy, acknowledging the potential bias (4). In contrast, a recent pooled analysis of two phase III trials in high-risk prostate cancer demonstrated that patients treated with radiotherapy had a lower incidence of distant metastasis (26).

A consistent finding across multiple studies is that older patients with more advanced disease are more likely to receive radiotherapy, whereas younger patients with less advanced disease are preferentially treated with surgery. This difference in baseline characteristics may partially explain the relatively higher all-cause mortality observed in radiotherapy series (27, 28). In summary, surgery and radiotherapy appear to offer comparable oncologic outcomes, particularly in early disease. Nevertheless, in high-risk disease, the outcomes are conflicting, largely due to inherent differences in clinical and disease characteristics and potential bias in patient selection.

Interestingly, we observed better BRFS in the radiotherapy group compared with surgery (90.5% vs. 59.8%), which could be related to the high percentage of positive margins (36%) in our surgical series. Although this rate exceeds those typically reported in the literature (20–27%) (29), it is likely attributable to the high incidence of extracapsular extension in our patients (46.1%), as several reports have shown that margin positivity can reach up to 44% in cases with T3 disease. Moreover, extracapsular extension was associated not only with higher rates of positive surgical margins but also with poorer biochemical control (30), which might have contributed to the inferior BRFS observed with surgery in our study, especially because we detected inferior BRFS with a positive margin compared to a negative one. Moreover, the routine use of ADT in conjunction with radiotherapy for intermediate- and high-risk patients likely contributed to improved biochemical control in this group. As previously mentioned, clinical trials have demonstrated that combined radiotherapy and long-term ADT significantly improve progression-free and overall survival in high-risk prostate cancer (31, 32).

A key observation from our analysis is that oncologic outcomes have remained stable, with slight improvements over time, despite the evolution of treatment techniques. OS was comparable across treatment eras, and CSS and BRFS showed modest gains in more recent years. These improvements might reflect advances in treatment delivery, including the introduction of IMRT and VMAT at our center. While such techniques are not expected to directly enhance oncologic outcomes, they may contribute to reduced toxicity and improved treatment tolerability (33, 34). Similarly, the adoption of laparoscopic and robotic surgery has been associated with fewer postoperative complications, but the effect on oncologic outcomes is controversial (35). Collectively, the gradual integration of more advanced treatment modalities appears to have resulted in modest improvements in disease control and survival, accompanied by better management of treatment-related complications. However, these observations warrant formal evaluation through cost-effectiveness analyses, particularly in financially constrained settings such as Jordan. In resource-limited environments, the management of localized prostate cancer should be guided by risk stratification, available expertise, and cost-effectiveness considerations, given the comparable survival outcomes observed between surgery and radiotherapy. Treatment selection should therefore depend on the availability of radiotherapy services or experienced surgeons in high-volume centers and be determined within a multidisciplinary framework, with greater centralization of care to optimize outcomes and ensure efficient use of limited resources.

While our study offers valuable insights, several limitations must be acknowledged. The retrospective design introduces a potential selection bias, particularly in treatment allocation. This was clear in the age and risk groups distribution between arms; these baseline confounders might have implications for the study results and reported outcomes. Therefore, our findings should be interpreted with caution, but these results may serve as hypothesis-generating data for future prospective randomized clinical trials or propensity score-matched analysis. Another major limitation of this study is the lack of detailed information on treatment-related toxicity, quality of life, and functional outcomes, including urinary, bowel, and sexual function. The absence of these data precludes a comprehensive comparison of the true patient-centered impact of radical prostatectomy and radiotherapy and limits their incorporation into shared decision-making for men with localized prostate cancer. Addressing this critical gap should be a priority for future prospective, multicenter research efforts in the region, with systematic collection of patient-reported outcomes to better inform treatment selection.

Despite these limitations, this study provides the first comparative evidence from the MENA region showing that both radical prostatectomy and radiotherapy can achieve excellent long-term oncologic outcomes in localized prostate cancer when delivered in a tertiary cancer center under the umbrella of multidisciplinary decision-making. Future efforts should focus on early screening and detection using novel tools such as MRI and PSMA, in addition to the integration of quality-of-life scores to capture real-world complications of each treatment modality.

## Conclusions

This study represents the first comprehensive comparison of radical prostatectomy and radiotherapy for localized prostate cancer in Jordan and, to our knowledge, in the MENA region. Both treatment modalities demonstrated excellent long-term oncologic outcomes, with no significant differences in overall or cancer-specific survival. Radiotherapy, particularly when combined with ADT, was associated with superior biochemical control, while surgery showed higher recurrence rates, largely influenced by adverse pathological features such as positive margins and extracapsular extension. Notably, despite evolving treatment techniques, oncologic outcomes have remained stable, with modest improvements in biochemical control observed over the years. These findings emphasize the importance of delivering care within a multidisciplinary approach. The treatment of localized prostate cancer should be individualized according to clinical and disease characteristics. Future prospective multicenter collaborations across the region are needed to validate these findings, assess patient-reported outcomes, and evaluate cost-effectiveness in resource-limited settings.

## Data Availability

The data supporting the findings of this study are available from the corresponding author upon reasonable request, without undue reservation.
