# Supplementary material for: Prostatectomy versus definitive radiation for localized prostate cancer: revisiting the debate at a tertiary cancer center
Source: Front Oncol. 2026 Mar 2;16:1756635. doi: 10.3389/fonc.2026.1756635 (PMC12989380; doi:10.3389/fonc.2026.1756635)
Supplement: Supplementary file 1 [file DataSheet1.docx]

**Supplementary documents**

**Supplementary Figure 1a:** Overall survival according to surgical margin status.

**Supplementary Figure 1b:** Biochemical recurrence–free survival according to surgical margin status.

**Supplementary Figure 1c:** Cancer-specific survival according to surgical margin status.

**Supplementary (2):** Multivariable Cox regression model evaluating predictors of biochemical recurrence–free survival.

| **Parameter** | **Variable** | **p-value** | **Hazard Ratio** | **95% Hazard Ratio Confidence Limits** | | **Overall p-value** |
| --- | --- | --- | --- | --- | --- | --- |
| Age group | age>70 vs. Age <=70 | 0.0737 | 0.525 | 0.259 | 1.064 | 0.0737 |
| Comorbidities | Yes vs. No | 0.1024 | 0.651 | 0.389 | 1.090 | 0.1024 |
| Risk group | Intermediate risk vs. High risk | <.0001 | 0.174 | 0.087 | 0.346 | <.0001 |
| Risk group | Low Risk vs. High risk | 0.9821 | 0.000 | NA | NA |  |
| treatment | Radiotherapy vs. Prostatectomy | <.0001 | 0.169 | 0.099 | 0.290 | <.0001 |

**Supplementary 3:** Multivariable Cox proportional hazards regression analysis for overall survival (OS).

| **Parameter** | **Variable** | **p-value** | **Hazard Ratio** | **95% Hazard Ratio Confidence Limits** | | **Overall**  **p-value** |
| --- | --- | --- | --- | --- | --- | --- |
| Age group | Age>70 vs. Age <=70 | 0.0274 | 1.704 | 1.061 | 2.736 | 0.0274 |
| Comorbidities | Yes vs. No | 0.0104 | 3.302 | 1.324 | 8.236 | 0.0104 |
| Year group | 2012-2017  vs. 2006-2011 | 0.0258 | 2.043 | 1.090 | 3.830 | 0.0647 |
| Year group | >=2018  vs. 2006-2011 | 0.0770 | 2.572 | 0.903 | 7.326 |  |
| Risk group | Intermediate risk  vs. high risk | 0.0702 | 0.604 | 0.350 | 1.042 | 0.0037 |
| Risk group | Low Risk vs. High risk | 0.0131 | 2.835 | 1.245 | 6.458 |  |

**Supplementary 4:** Adjusted hazard ratio for N stage in cancer-specific survival using multivariable Cox regression.

| **Parameter** |  | **p-value** | **Hazard Ratio** | **95% Hazard Ratio Confidence Limits** | |
| --- | --- | --- | --- | --- | --- |
| N Stage | N1 vs. N0 | 0.0062 | 5.255 | 1.600 | 17.259 |

**Supplementary Figure 5a:** Overall survival stratified by nodal status (N0 vs N1)

**Supplementary Figure 5b:** Biochemical recurrence-free survival stratified by nodal status (N0 vs N1)

**Supplementary Figure 5c:** Cancer-specific survival stratified by nodal status (N0 vs N1)

**Supplementary 6a:** Overall survival stratified by treatment era

**Supplementary 6b:** Biochemical recurrence-free survival stratified by treatment era

**Supplementary Figure 6c:** Cancer-specific survival stratified by treatment era
